# Fenofibrate inhibits the expression of VEGFC and VEGFR-3 in retinal pigmental epithelial cells exposed to hypoxia

**DOI:** 10.3892/etm.2015.2697

**Published:** 2015-08-21

**Authors:** JIANFENG ZHAO, YU GENG, HAIRONG HUA, BIYUN CUN, QIANBO CHEN, XIAOTING XI, LIUSHU YANG, YAN LI

**Affiliations:** Department of Ophthalmology, The First Affiliated Hospital of Kunming Medical University, Kunming, Yunnan 650031, P.R. China

**Keywords:** human retinal pigment epithelial cells, human umbilical vein endothelial cells, hypoxia, fenofibrate, vascular endothelial growth factor C, vascular endothelial growth factor receptor-3

## Abstract

The aim of the present study was to examine the mechanisms through which fenofibrate inhibits the ability of human retinal pigment epithelial cells (RPE cells) exposed to hypoxia to stimulate the proliferation and migration of human umbilical vein endothelial cells (HUVECs). For this purpose, RPE cells and HUVECs were divided into the following groups: RPE-normoxia, RPE + fenofibrate, RPE-hypoxia, RPE hypoxia + fenofibrate; HUVECs normal culture and HUVECs + RPE-hypoxia culture supernatant. RPE cell hypoxia was induced by cobalt(II) chloride (CoCl_2_). A superoxide anion probe was used to measure the production of superoxide anion, which is indicative of hypoxic conditions. Cell proliferation was assessed by MTT assay, and the expression of vascular endothelial growth factor C (VEGFC) and vascular endothelial growth factor receptor-3 (VEGFR-3) in the RPE cell culture supernatant was measured by enzyme-linked immunosorbent assay (ELISA). The migration ability of the HUVECs was determined by scratch-wound assay, and the angiogenic ability of the HUVECs was examined by measuring cell lumen formation. The mRNA and protein expression levels of VEGFC and VEGFR-3 in the RPE cells were measured by RT-qPCR and western blot analysis, respectively. Our results revealed that fenofibrate inhibited the increase in the expression and release of VEGFC and VEGFR-3 into the RPE cell culture supernatant induced by exposure to hypoxia. The culture of HUVECs in medium supernatant of RPE cells epxosed to hypoxia enhanced the viability and migration ability of the HUVECs and promoted lumen formation; these effects were inhibited by fenofibrate. In conclusion, our data demonstrated that the exposure of RPE cells to hypoxia induced the expression and release of VEGFC and VEGFR-3 into the cell culture supernatant. The culture of HUVECs in conditioned medium from RPE cells exposed to hypoxia increased VEGFC and VEGFR-3 expression, and promoted the proliferation and migration of the HUVECs, as well as capillary tube formation, suggesting that RPE cells play an important role in the formation of choroidal neovascularization resulting from hypoxia. Fenofibrate inhibited the upregulation of VEGFC and VEGFR-3 in the RPE cells exposed to hypoxia, and thus reduced the ability of HUVECs to form new blood vessels.

## Introduction

Choroidal neovascularization (CNV) is a pathological neovascularization that affects choroids, as these new vessels have high permeability, structural defects, and a high susceptibility to bleeding and seepage. Such defective vasculature can lead to the formation of scars, which can greatly affect visual function (1); thus, CNV is one of the leading causes of blindness. The pathogenesis of CNV is not yet completely understood and studies have indicated that retinal pigment epithelial cells (RPE cells) exposed to hypoxia play an important role in the development of CNV (2). RPE cells are located between the neural retina and choroids. In normal eyes, the retinal pigment epithelium (RPE), together with Bruch's membrane and choriocapillaris, form a complex. During CNV, choroidal capillary endothelial cells of the complex keep dividing and proliferating continuously, forming new blood vessels and entering into the site between Bruch's membrane and the RPE layer, or between the RPE layer and the neurosensory retina (3). It has been suggested that in the RPE, hypoxia is caused by the reduction of choroid local blood flow and the oxygen diffusion barrier, which ranges from choroids to the RPE and neuroepithelium. Hypoxic RPE cells secrete large amounts of cytokines and factors that induce angiogenesis, such as vascular endothelial growth factor (VEGF), basic fibroblast growth factor (bFGF), as well as transforming growth factor (TGF), angiopoietin (Ang), and receptor tyrosine kinases Tie1 and Tie2 (4,5). VEGF plays an important role in angiogenesis and leakage and is involved in all stages of diabetic retinopathy (6). It has been demonstrated that the expression of VEGF in RPE cells, retinal endothelial cells, pericytes and Müller cells increases under disease conditions (5). Hypoxia induces the production of hypoxia-inducible factor (HIF), which elevates the transcription and stability of VEGF and VEGF receptor (VEGFR), and enhances the biological effects of VEGF. When normoxia is restored, the *VEGF* mRNA levels decrease to baseline levels (7). Thus, by controlling the activity of RPE cells, it may be possible to inhibit the induction of VEGF and attenuate the formation of new blood vessels.

It is known that the VEGFA/VEGFR-2 axis is a key regulated signaling pathway of angiogenesis (8). In vascular endothelial cells, the presence of VEGFR-2 along with its ligand, VEGFA, promote endothelial cell mitosis and chemotactic response, thus leading to the formation of new blood vessels (9). Drugs such as ranibizumab, bevacizumab, pegaptanib, as well as others block the VEGFA/VEGFR-2 axis and inhibit the development of CNV. These drugs have achieved some therapeutic effects, which has provided hope for the treatment of retinopathy and CNV. However, these drugs only have a single application point (single point of action) and their therapeutic effects are limited (10). It has been demonstrated that VEGF inhibitors, although effective during the early stages of treatment, gradually lose effectiveness due to the development of drug resistance, and do not effectively inhibit angiogenesis in long-term therapy (11). However, the inhibition of VEGF alone may lead to the activation of other types of pro-angiogenic factors released from histiocytes, which can also promote angiogenesis (12). Therefore, it is necessary to further explore other signaling pathways of angiogenesis as therapeutic targets.

It has previously been suggested that VEGFR-3 is only associated with the formation of lymphatic vessels (13). However, it has also been found that embryonic VEGFR-3 is also involved in angiogenesis (14). VEGFR3 expression is confined to the lymphatic vasculature in benign lesions; however, its expression increases during wound healing and tumor angiogenesis (15,16). As shown in the study by Yuasa *et al*, the expression of VEGFR-3 may be a suitable biomarker to predicet response to renal disease (17), which suggests that the activation of VEGFR-3 plays an important role in angiogenesis (3). Tammela *et al* reported that the inhibition of VEGFR-3 with a monoclonal antibody reduced vascular sprouting, vascular branches and endothelial cell proliferation during embryonic development and tumor growth (14), which suggests that VEGFR-3 is a novel target in the treatment of CNV.

Fenofibrate is a common lipid-lowering drug, which reduces plasma triglyceride and low-density lipoprotein cholesterol levels, and increases high-density lipoprotein cholesterol levels in patients with hyperlipidemia. Apart from its lipid-lowering effects, fenofibrate has several other effects, such as the improvement of vascular endothelial function, anti-inflammatory and antioxidant effects and the inhibition of angiogenesis (18–20). In recent years, fibrate lipid-lowering drugs have been studied extensively regarding diabetic retinal neovascularization (21–23); however, to the best of our knowledge, there is no information available to date on their effects on CNV. Researchers have focused on the association between VEGFA-VEGFR-2 and neovascular disease (24), but not on VEGFC-VEGFR-3. Thus, in the present study, we examined the mechanisms of action of fenofibrate using an RPE cell model of hypoxia, in an aim to determine whether fenofibrate exerts an effect on RPE cells to influence the secretion of VEGFC, thus altering the function of endothelial cells.

## Materials and methods

### 

#### Reagents and kits

Dulbecco's modified Eagle's medium (DMEM) containing L-glutamine, fetal bovine serum (FBS), penicillin-streptomycin and 0.25% pancreatin-ethylenediaminetetraacetic acid (EDTA) were all purchased from Gibco/Invitrogen, Grand Island, NY, USA. ECA medium was obtained from ScienCell (San Diego, CA, USA); the cell culture plate was from Corning Inc., Corning, NY, USA; the 0.22 µm disposable filter was obtained from Millipore (Billerica, MA, USA); MTT reagent and cobalt(II) chloride (CoCl_2_) were both from Aladdin Reagent (Shanghai) Co., Ltd., Shanghai, China; the superoxide anion probe was from Beyotime, Shanghai, China; fenofibrate was obtained from Sigma Chemical Co., St. Louis, MO, USA; Matrigel was purchased from BD Biosciences, Bedford, MA, USA; anti-VEGFR-3 antibody (20712-1-AP) was from Proteintech, Chicago, IL, USA; anti-VEGFC antibody (sc-9047) was from Santa Cruz Biotechnology, Inc., Dallas, TX, USA; anti-glyceraldehyde 3-phosphate dehydrogenase (GAPDH) antibody (P30008) and IgG-HRP (M21002) were from Abmart (Shanghai, China); PVDF membranes were purchased from Millipore; skim milk was purchased from BD Biosciences; HRP-substrate coloring solution was obtained from Millipore; the VEGFC enzyme-linked immunosorbent assay (ELISA) kit and the VEGFR-3 ELISA kit were from USCN Life Science, Inc., Wuhan, China.

#### Cell culture

RPE cells (CRL-2302) were obtained from ATCC (Manassas, VA, USA) and human umbilical vein endothelial cells (HUVECs) were from ScienCell. The RPE cell model of hypoxia was established as follows: the RPE cells were cultured in a 37°C, 5% CO_2_ saturated humidity incubator with DMEM (containing 1% penicillin-streptomycin and 10% FBS). The cells were grown to 60–70% cell density in DMEM containing 1% FBS for 24 h, and the medium was then changed to 1% FBS DMEM containing 200 µmol/l CoCl_2_, for the induction of hypoxia. Following culture for 24 h, the old medium was replaced with DMEM (with 1% FBS) containing 100 µmol/l fenofibrate + 200 µmol/l CoCl_2_ and culture was continued for 24 h. Cells in the control group were treated as follows: the cells were cultured in DMEM (with 1% FBS) for 48 h, followed by culture in DMEM (with 1% FBS) containing 200 µmol/l CoCl_2_ for 48 h, and then with DMEM (with 1% FBS) containing 100 µmol/l fenofibrate for 24 h. Cell culture was conducted in in a 37°C and 5% CO_2_ saturated humidity incubator. Following treatment with CoCl_2_ for 48 h, a superoxide anion probe was used to measure the production of superoxide anion, which is indicative of hypoxic conditions. The HUVECs were cultured in a 37°C and 5% CO_2_ saturated humidity incubator with extracellular matrix (ECM) medium (with 1% penicillin-streptomycin, 10% FBS). The HUVECs were cultured with RPE culture supernatant that was collected following the exposure of the RPE cells to hypoxia, and the control cells were cultured in DMEM (with 1% FBS). There were 8 samples in each group.

#### MTT assay

The cells were treated as described above. The cells were seeded in a 96-well culture plate with 1×10^4^ cells/well, and the following day, the cells were treated according to their grouping. Following culture for 48 h, the culture medium was removed and 100 µl MTT solution were added, followed by incubation for 4 h in an incubator at 37°C; the MTT solution was then removed and 100 µl DMSO were added to each well, followed by mixing for 10 min, and the OD value was then measured at 570 nm using a spectral scanning multimode reader (Varioskan Flash, Thermo Fisher Scientific Inc., Waltham, MA, USA).

#### ELISA

Following subculture for 48 h, the RPE cells were divided into 4 groups: group 1, CoCl_2_-induced hypoxia for 48 h; group 2, exposure to CoCl_2_ for 48 h and 100 µmol/l fenofibrate treatment for 24 h; group 3, treatment with 100 µmol/l fenofibrate for 24 h; group 4, the control group was treated with DMEM (with 1% FBS). The supernatant was then collected; 8 samples were taken from each group. Following filtration with a 0.22 µm filter, ELISA was used to detect VEGFC and VEGFR-3 protein expression in the supernatant.

#### RT-qPCR

The RPE cells were divided into the following groups: group 1, CoCl_2_-induced hypoxia for 48 h; group 2, exposure to CoCl_2_ for 48 h and fenofibrate treatment for 24 h; group 3, treatment with fenofibrate for 24 h; group 4, the control cells were treated with DMEM containing 1% FBS. Following treatment, TRIzol reagent was used to extract the total RNA. The RevertAid First Strand cDNA synthesis kit (Thermo Fisher Scientific Inc.) was used for the synthesis of first strand of cDNA by reverse transcription. The PCR conditions were as follows: cDNA template 1 µl, forward primer 0.5 µl, reverse primer 0.5 µl (Table I), FastStart Universal SYBR-Green Master (Rox; Roche Life Science, Branford, CT, USA) 10 µl, nuclease-free water 8 µl, mixing, total volume of 20 µl, in 96-well PCR plates; triplicate wells for each gene in each sample were taken, qPCR was carried out using an ABI 7300 qPCR instrument (Applied Biosystems, Foster City, CA, USA). PCR reactions were carried out as follows: stage 1: 95°C, 3 min; stage 2 (for 40 cycles): step 1, 95°C, 15 sec; step 2, 60°C, 30 sec. Melting curve analysis was carried out as follows: 95°C: 15 sec; 60°C: 30 sec; 95°C: 15 sec. All experiments were repeated 3 times. The 2^−ΔΔCt^ method was used to analyze the results.

#### Western blot analysis

The RPE cells were grouped in the same manner as in RT-qPCR. Following digestion with 0.25% trypsin, total protein was extracted from the cells in each group using RIPA buffer and subjected to SDS-PAGE electrophoresis and transferred onto PVDF membranes. The PVDF membranes were blocked with 5% skim milk at room temperature for 1 h, followed by incubation with anti-VEGFC and anti-VEGFR-3 antibodies overnight at 4°C (VEGFR-3, 1:200; VEGFC, 1:500), and then with HRP-labeled universal secondary antibody (1:2,000, Abmart) at room temperature for 2 h. GAPDH antibody (1:1,000) was used as a loading control. The protein bands were visualized with HRP-substrate coloring solution for 1 min and ImageJ software was used to analyze the density of the bands and protein quantity.

#### Wound healing assay

*In vitro*, the scratch-wound assay was used to detect the migration of HUVECs, as previously described (25). The HUVECs were seeded in a 6-well culture plate, and when the cells had attached completely, a vertical straight line was drawn in each well using a 10 µl aseptic suction head, by scraping the cells. The medium was then removed and the cells were washed 2–3 times with phosphate-buffered saline (PBS), followed by incubation with the culture supernatant from the RPE cells exposed to CoCl_2_-induced hypoxia. The control HUVECs were incubated in DMEM (with 1% FBS). They were cultured in a 37°C, 5% CO_2_ incubator. A total of 8 samples was taken from each group and images were captured under a microscope (DMI3000 B, Leica Microsystems, Mannheim, Germany) at x200 magnification, at 24 and 0 h after scratching. ImageJ software was used to measure the wound width.

#### Cell lumen formation

The cells were plated with Matrigel, and the HUVECs were stimulated to form capillary-like lumen, as previously described (26). Matrigel (60 µl) was added to a well of a 96-well plate, and the culture plate was shaken gently. Following Matrigel solidification, a 100 µl suspension of HUVECs was added to each well at a cell density of 1×10^4^. The cells were then treated with the supernatant from RPE cells exposed to hypoxia, and cultured in a 37°C, 5% CO_2_ incubator for 48 h. Vwssel lumen formation was observed at x50 magnification and counted (at least 5 horizons were randomly selected, counted and averaged; 8 samples were taken from each group). The number of lumen formed represented the angiogenic ability of the HUVECs.

#### Statistical analysis

Data are presented as the means ± standard deviation (SD). SPSS 18.0 software (SPSS. Inc., Chicago, IL, USA) was used for statistical analysis. One-way analysis of variance was used to make comparisons between groups, and the t-test was used for comparisons between 2 groups. A value of P<0.05 was considered to indicate a statistically significant difference.

## Results

### 

#### CoCl_2_-induced hypoxia in RPE cells

**Figure 1.**
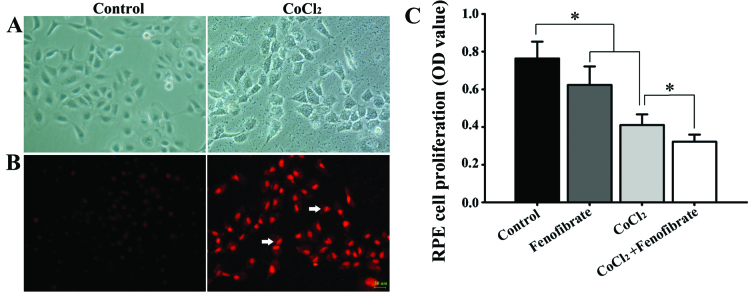
Effects of hypoxia induced by CoCl_2_ and fenofibrate on retinal pigment epithelial cells (RPE cells). (A) Normal RPE cells (left panel) and RPE cells exposed to CoCl_2_ to induce hypoxia (right panel). (B) The hypoxia-induced state of RPE cells was detected using a superoxide anion probe to measure superoxide anion production in normal RPE cells (left panel) and RPE cells exposed to CoCl_2_ to induce hypoxia (right panel). After 48 h, the morphology of the RPE cells had changed. Compared with the control group, the cell body became round in the treated cells and the cell volume became enlarged [(A) right column]. A large number of superoxide anions in the RPE cells could be observed [(B) right column, arrows]. (C) Effects of fenofibrate on RPE cell proliferation. Exposure to CoCl_2_ and treatment with fenofibrate reduced the proliferation of RPE cells and the addition of fenofibrate following CoCl_2_-induced hypoxia in RPE cells further suppressed cell proliferation. *p<0.05.

The RPE cells were treated with CoCl_2_ for 48 h to induce chemical hypoxia. Compared with the control group, the cell body became round in the treated cells, the volume became enlarged (Fig. 1A), the cell growth rate was reduced, and the production of superoxide anion was increased (Fig. 1B). Cell viability decreased after the RPE cells were epxosed to hypoxia. The addition of fenofibrate to the normal RPE cells led to a decrease in cell viability. Treatment of the hypoxic RPE cells with fenofibrate decreased the cell viability even further (Fig. 1C).

#### VEGFC and VEGFR-3 protein levels in the RPE cell supernatant

**Figure 2.**
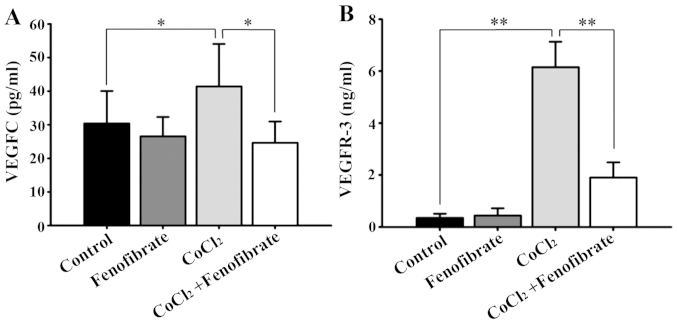
The levels of vascular endothelial growth factor C (VEGFC) and VEGF receptor-3 (VEGFR-3) in the culture supernatant of retinal pigment epithelial cells (RPE cells). (A and B) The contents of VEGFC and VEGFR-3 in the culture medium of RPE cells in the different groups. Results are the means ± SD (n=8). VEGFC was detected in the supernatant of normal RPE cells, but the levels of VEGFR-3 were quite low. Compared with the normal group, fenofibrate had little effect on the synthesis and secretion of VEGFC and VEGFR-3 in RPE cells. Following the induction of hypoxia by CoCl_2_ in RPE cells, the synthesis and secretion of VEGFC and VEGFR-3 was significantly increased, but after the addition of fenofibrate and incubation for 24 h, the synthesis and secretion of VEGFC and VEGFR-3 was significantly downregulated in the RPE cells exposed to hypoxia. *p<0.05 and **p<0.01.

The normal RPE cells expressed VEGFR-3 at low levels, but expressed a detectable amount of VEGFC. Following CoCl_2_-induced hypoxia, the amount of VEGFC and VEGFR-3, which was synthesized and secreted by the RPE cells into the cell culture medium, increased significantly (Fig. 2). Fenofibrate had little effect on the expression of VEGFC and VEGFR-3 in the normal RPE cells; however, it significantly decreased the expression of VEGFC and VEGFR-3 in the hypoxic RPE cells (Fig. 2).

#### Effects of fenofibrate on VEGFC and VEGFR-3 expression in hypoxic RPE cells

**Figure 3.**
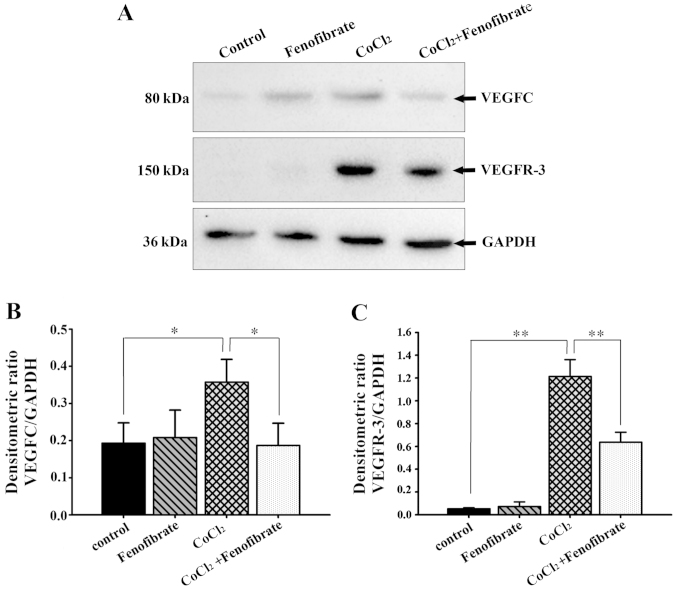
Western blot analysis of vascular endothelial growth factor C (VEGFC) and VEGF receptor-3 (VEGFR-3) expression in retinal pigment epithelial cells (RPE cells). (A) Western blots representing each group: VEGFC (80 kDa), VEGFR-3 (150 kDa) and glyceraldehyde 3-phosphate dehydrogenase (GAPDH) (36 kDa). (B) Relative expression of GAPDH and VEGFC in RPE cells. (C) Relative expression of GAPDH and VEGFR-3 in RPE cells. Results shown are the means ± SD; n=8. VEGFC was expressed in normal RPE cells, whereas VEGFR-3 was not. Compared with the control group, the effects of fenofibrate on VEGFC and VEGFR-3 expression in normal RPE cells were not significant. The intracellular VEGFC and VEGFR-3 expression in RPE cells after CoCl_2_-induced hypoxia was significantly increased, and fenofibrate significantly reduced the synthesis of VEGFC and VEGFR-3 in RPE cells following exposure to hypoxia. *p<0.05 and **p<0.01.

**Figure 4.**
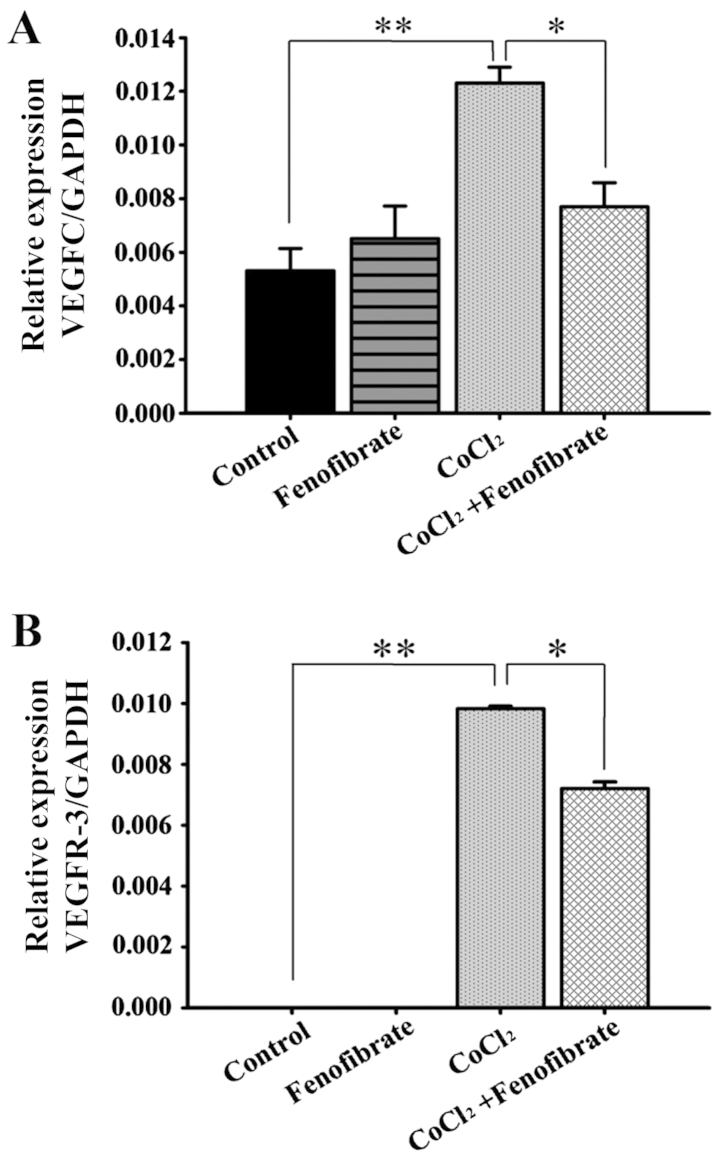
mRNA expression levels of vascular endothelial growth factor C (*VEGFC*) and VEGF receptor-3 (*VEGFR-3*) in retinal pigment epithelial cells (RPE cells) analyzed by RT-qPCR. (A and B) Relative mRNA expression of of *VEGFR-3* and *VEGFC* in RPE cells in each group. *VEGFC* and *VEGFR-3* mRNA were compared to glyceraldehyde 3-phosphate dehydrogenase (GAPDH) in RPE cells of each group. The 2^−ΔΔCt^ method was used to analyze the results. The data are expressed as the means ± SD, n=8. In normal RPE cells, *VEGFC* was expressed and *VEGFR-3* was not. The expression of *VEGFC* and *VEGFR-3* in normal RPE cells was not significantly affected by fenofibrate. Following exposure to hypoxia induced by CoCl_2_, the expression of *VEGFC* and *VEGFR-3* in RPE cells was upregulated. Both *VEGFR-3* and *VEGFC* were downregulated after the addition of fenofibrate. *p<0.05 and **p<0.01.

The expression levels of VEGFC and VEGFR-3 in the cells that were cultured under hypoxic conditions with or without fenofibrate, were measured by western blot analysis and RT-qPCR. In comparison to the housekeeping gene, GAPDH, we calculated the relative expression and found that there were detectable levels of VEGFC expression in the normoxic RPE cells, but very low levels of VEGFR-3 protein expression (Fig. 3A). The expression of both VEGFC and VEGFR-3 in the RPE cells increased following the induction of hypoxia for 48 h; the increase in VEGFR-3 expression was more significant. Following exposure to hypoxia, the addition of fenofibrate and further incubation for 24 h significantly decreased the expression of VEGFC and VEGFR-3 in the RPE cells (Fig. 3B and C). The changes in the *VEGFC* and *VEGFR-3* mRNA levels (Fig. 4) were consistent with the trends observed with the protein levels, determined by western blot analysis (Fig. 3).

#### Effects of culture with the culture supernatant of hypoxic RPE cells on HUVEC proliferation, migration and tube formation

**Figure 5.**
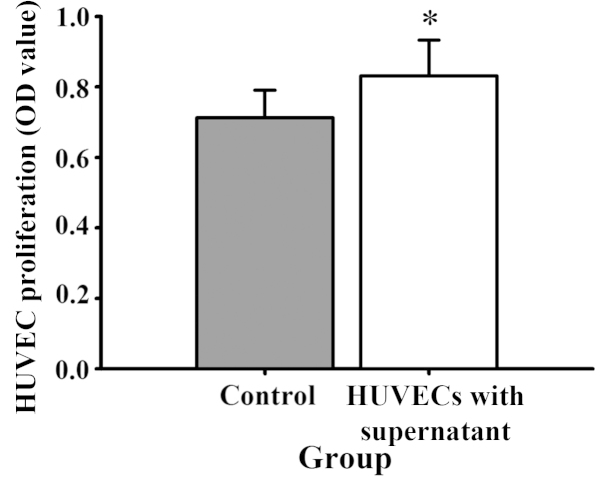
Effects of culture with the culture supernatant of retinal pigment epithelial cells (RPE cells) on the proliferation of human umbilical vein endothelial cells (HUVECs). By MTT assay, we detected the effects of RPE cell culture medium on the proliferation activity of HUVECs. Control group: HUVECs in the control group were cultured with Dulbeccos modified Eagles medium (DMEM) containing 1% fetal bovine serum (FBS). HUVECs cultured with RPE cell medium: HUVECs were cultured in the culture supernatant of RPE cells exposed to hypoxia. Results shown are the means ± SD; n=8. Compared with the normal control group, culture with the supernatant of RPE cells exposed to hypoxia significantly increased the proliferation of the HUVECs.

**Figure 6.**
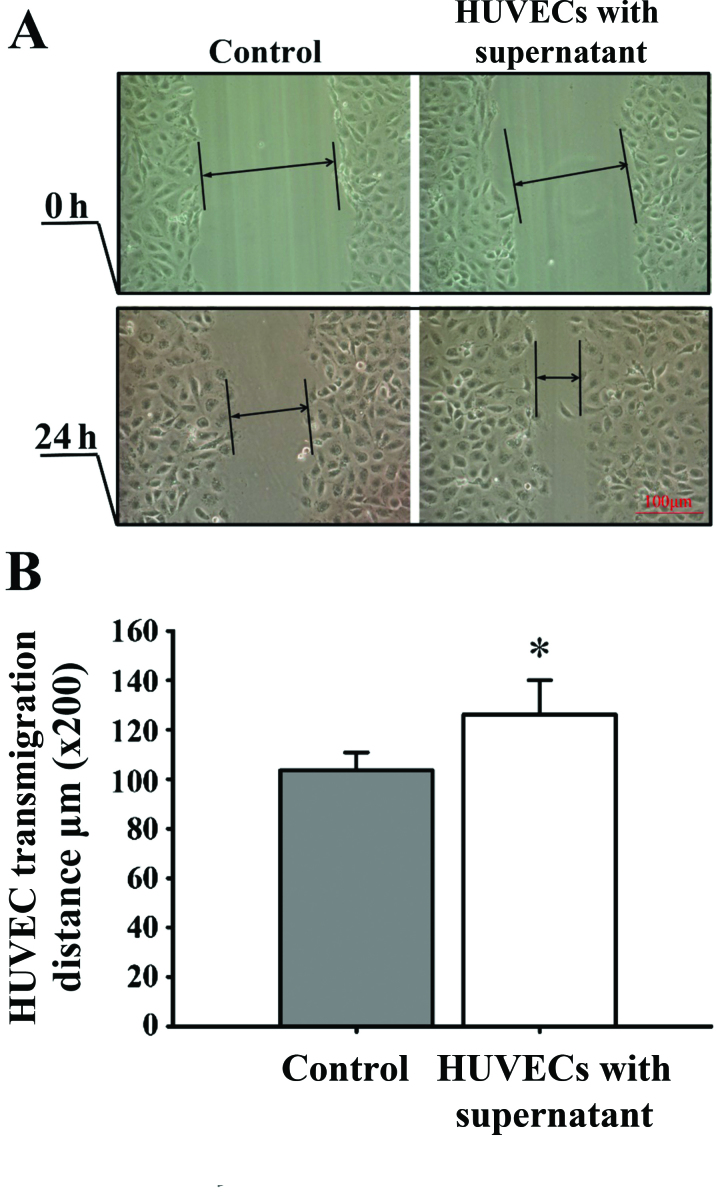
Analysis of the migration of human umbilical vein endothelial cells (HUVECs) by scratch-wound assay. (A) Migration of HUVECs cultured for 24 h in the supernatant of control retinal pigment epithelial cells (RPE cells) (left panel) and in the supernatant of RPE cells epxosed to hypoxia (right panel) (x200 magnification). (B) HUVEC migration rate. The scratch width of each group was 200 µm, and the cell migration distance of the cells between the scratch edges was observed after 24 h. Results shown are the means ± SD; n=8. Compared with the control group, cutlure with the supernatant from hypoxic RPE cells significantly increased the HUVEC migration rate. *p<0.05.

**Figure 7.**
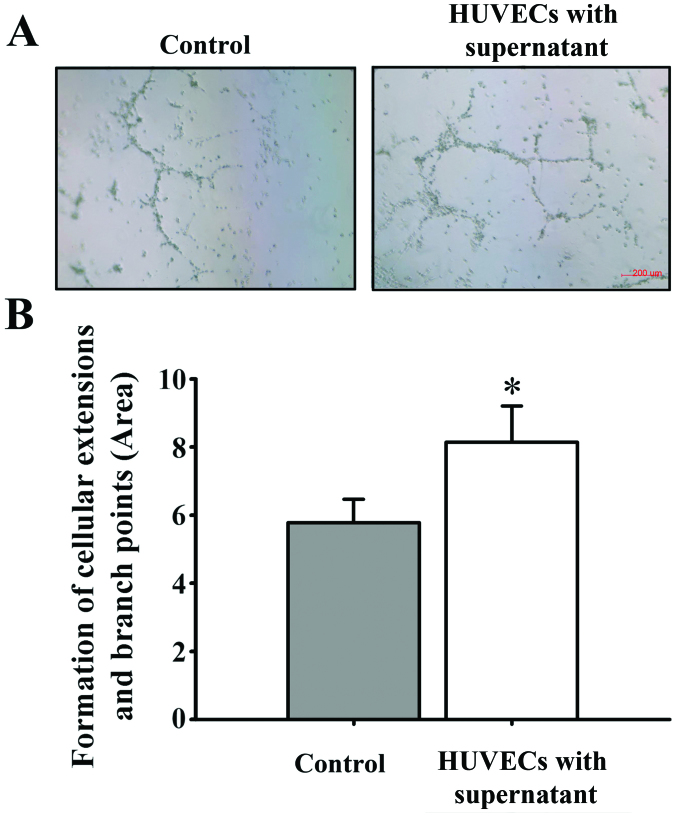
Effects of culture with the supernatant of retinal pigment epithelial cells (RPE cells) on angiogenic activity of human umbilical vein endothelial cells (HUVECs). Cultured HUVEC cells were divided into 2 groups and plated in Matrigel. The control group was treated with Dulbecco's modified Eagle's medium (DMEM) containing 1% fetal bovine serum (FBS). The experimental group was treated with the supernatant of RPE cells exposed to hypoxia. (A) Control group (left panel) and the experimental group (right panel) after 48 h of incubation (x50 magnification). (B) Area covered by cellular extensions linking HUVEC masses and branch points. These calculations were performed on images of standardized fields from at least 5 wells/experimental condition. Results are the means ± SD, with 8 samples in each group. Compared with the control group, culture with the supernatant of RPE cells exposed to hypoxia significantly increased the tube formation ability of the HUVECs. *p<0.05.

Cell culture medium obtained from the cultures of hypoxic RPE cells at 48 h, was used to culture the HUVECs directly. By MTT assay, scratch-wound assay and tube formation assay, the effects of hypoxia-conditioned cell supernatant on the angiogenic activity of the HUVECs were determined. The results revealed that compared with the control group, 48 h after the addition of the culture medium, HUVEC proliferation (Fig. 5), mobility (Fig. 6) and tube formation ability (Fig. 7) were significantly enhanced.

## Discussion

The damage to and destruction of the RPE-Bruch's membrane-choriocapillaris complex forms the anatomical basis for the formation of CNV (27). The changes in the extracellular microenvironment *in vivo*, promote the formation of neovascularization. Studies have demonstrated that there are three main reasons for the formation of intraocular neovascularization: hypoxia, inflammation and oncogene products (28,29). Neovascularization often occurs to meet the needs of physiology and metabolism of local tissues. VEGF is the most powerful cytokine known thus far, that can promote the formation of new vessels and it is considered that hypoxia induces the release of adenosine in tissue, which binds with its receptor and stimulates endothelial cells to synthesize VEGF. Recent studies have demonstrated that hypoxia induces the upregulation of intracellular HIF1 (30–32), which regulates the expression of a number of hypoxia stress proteins and upregulates VEGF, as well as many angiogenic factors in tumor tissues, and thus promotes the formation of new vessels. The process of choroid neovascularization has a close association with the functional changes of the RPE. VEGF expression has been detected in the RPE in animals, and VEGF expression is closely related to the development of CNV (33).

VEGFC belongs to the vascular endothelial growth factor/platelet derived growth factor (VEGF/PDGF) family, and VEGFR-3 is the receptor of VEGFC, which can only bind with VEGFC (or VEGFD). In the physiological state, VEGFR-3 only exists in the human body in lymphatic vessels and in some reticular endothelial cells. However, under pathological conditions, it plays an important role in promoting angiogenesis, such as tumor growth and wounding (34). During the course of embryonic development, the deletion of VEGFR-3 has been shown to lead to the developmental failure of the cardiovascular system, which suggests that VEGFR-3 plays a key role in the formation of blood vessels (35). Previous research on angiogenesis has focused on VEGF and its receptor, VEGFR-2, and research on the inhibition of angiogenesis has mainly focused on blocking the VEGF/VEGFR-2 signaling pathway (36). Tammela *et al* (14) found that silencing VEGFR-3 expression reduces vascular sprouting during tumor and embryonic development. The expression of VEGF and its receptors has also been found in mammalian RPE cells and in the ECM (37). To the best of our knowledge, the expression of VEGFC and its receptor, VEGFR-3, in RPE cells has not been reported to date. The present study demonstrated that normal RPE cells cultured *in vitro* expressed VEGFC, but not VEGFR-3. After the RPE cells were exposed to hypoxia *in vitro*, the expression of VEGFC and VEGFR-3 increased, which suggests that under pathological conditions, particularly when tissues are subjected to ischemia and hypoxia, the expression of VEGFC and VEGFR-3 is upregulated in RPE cells, and thus VEGFC and VEGFR-3 can be secreted into the extracellular medium. We found that HUVEC proliferation and tube formation increased when the cells were cultured in the culture supernatant from RPE cells exposed to hypoxia, which contained high levels of VEGFC and VEGFR-3 expression. However, whether VEGFC and VEGFR-3 have a direct effect on HUVECs, or whether RPE cells can also express other VEGF members remains unknown (33,38). It can be inferred that RPE cells play an important role in the development of CNV. Moreover, the upregulation of VEGFC and VEGFR-3 is closely related to the development of CNV.

Fenofibrate is a lipid-regulating drug, which is an agonist of the peroxisome proliferator-activated receptor (PPAR) (39). In addition to its hypolipidemic effects, fenofibrate also inhibits angiogenesiss. It has been demonstrated that fenofibrate inhibits the proliferation of capillary endothelial cells induced by bFGF and the proliferation and migration activity of HUVECs induced by VEGF (40). At the same time, fenofibrate downregulates VEGFR-2 expression in HUVECs, thus inhibiting the formation and development of neovascularization (41). It has been found that fenofibrate inhibits the formation of micro-blood vessels through various mechanisms, such as downregulating SP1 activity, blocking the signaling pathway of VEGF and Wnt, and inhibiting the proliferation of vascular endothelial cells and capillary tube formation (42). Although it has been found that fenofibrate has some potential effects on vascular formation and protection, our knowledge on its effects on RPE cells, which play an important role in the development of CNV, is still limited. In this study, we found that fenofibrate downregulated the expression of VEGFC and VEGFR-3 in the RPE cells in a hypoxic environment. Thus, we hypothesized that fenofibrate inhibits the development of CNV through the downregulation of VEGFC and VEGFR-3 in RPE cells. It has been previously demonstrated that fenofibrate inhibits the activation of HIF-1, and this results in the downregulation of VEGF (43). Thus, it possible that fenofibrate downregulates VEGFC expression through the same pathway. However, in our experiments, the level of free VEGFR-3 was elevated in the culture supernatant, indicating that for RPE cells, VEGFR-3 can also be released in to the extracellular environment, and that fenofibrate can downregulate the expression of VEGFR-3 in the supernatant. In this study, after the HUVECs were cultured with the culture supernatant from hypoxic RPE cells, the expression of VEGFR-3 was significantly increased, and cell proliferation and tube formation were significantly enhanced. We hypothesized that although the expression of other VEGFs is increased in RPE cells following epxosure to hypoxia, the upregulation of the VEGFC receptor, VEGFR-3, confirmed that the expression of VEGFR-3 in HUVECs increases when the extracellular expression of VEGFC increases. It is possible that VEGFC plays a significant role in the development of CNV. Tammela *et al* (14) found that blocking the VEGFR-3 signaling pathway significantly reduced the number of capillary branches and sprouting in vascular endothelial cells (14). Currently, to the best of our knowledge, no reports are available indicating that fenofibrate inhibits the VEGFR-3 pathway, and thus, we hypothesized that fenofibrate inhibits the development of CNV mainly through the downregulation of VEGFC.

In conclusion, in the present study, we found that under hypoxic conditions, the expression of VEGFC and that of its receptor, VEGFR-3, was upregulated in RPE cells and that VEGFC and VEGFR-3 were secreted into the ECM. VEGFC increased the expression of VEGFR-3 in HUVECs. Treatment with fenofibrate decreased the expression of VEGFC and VEGFR-3 in the RPE cells, thereby suppressing HUVEC proliferation and capillary tube formation, which were induced by culture with the superntant of hypoxic RPE cells. Thus, it appears that treatment with fenofibrate may provide a new treatment strategy with which to prevent the development of CNV.

## Figures and Tables

**Table I.** Primers used for RT-qPCR.

Human gene	Forward primer	Reverse primer
*VEGFC* (103 bp)	5′-GTGTCCAGTGTAGATGAA-3′	5′-CCTGTTCTCTGTTATGTTG-3′
*VEGFR-3* (123 bp)	5′-GAGGGAAAGAATAAGACT-3′	5′-GGTCACATAGAAGTAGAT-3′
*GAPDH* (80 bp)	5′-AAAGGGTCATCATCTCTG-3′	5′-GCTGTTGTCATACTTCTC-3′

VEGFC, vascular endothelial growth factor C; VEGFR-3, vascular endothelial growth factor receptor-3; GAPDH, glyceraldehyde 3-phosphate dehydrogenase.
